# Evidence of glucose absorption in a neoformed intestine

**DOI:** 10.1007/s13304-022-01241-5

**Published:** 2022-01-20

**Authors:** Massimiliano Tuveri, Salvatore Paiella, Federico Boschi, Claudio Luchini, Giampaolo Perri, Clizia Gasparini, Alex Aresta, Aldo Scarpa, Roberto Salvia, Claudio Bassi

**Affiliations:** 1 General and Pancreatic Surgery Unit, Pancreas Institute, University of Verona, P.le L.A. Scuro n° 10, 37134 Verona, Italy; 2 Department of Computer Science, University of Verona, Verona, Italy; 3 Section of Pathology, Department of Diagnostics and Public Health, Pancreas Institute, University of Verona, Verona, Italy; 4 Radiology Unit, Pancreas Institute, University of Verona, Verona, Italy; 5 ARC-Net Research Center, University of Verona, Verona, Italy

**Keywords:** Short bowel surgery, Neointestine, Biological scaffolds, Glucose absorption

## Abstract

Recent advances in the field of tissue regeneration are offering promising therapeutic options for the treatment of short bowel syndrome. This study aimed to evaluate the glucose absorptive capacity of a neoformed intestine obtained from a biological scaffold in a rodent model and the steadiness of the engrafted segment area. Twenty-four male Sprague–Dawley rats were used for this study. Under anesthesia, a patch of biological material (2.2 × 1.5 cm) was engrafted in the anti-mesenteric border of the small bowels of 12 rats. Twelve rats were sham-operated. Animals were studied at 4, 8, and 10 months postengraftment. Functional and histological analyses were performed. The functional analysis was performed using an 18F-FDG analog as a probe and the results were acquired with an optical imager. The intensity of the fluorescent signal emitted by the neointestine was comparable with that emitted by the native intestine in all animals and was visible after injection in the preserved mesentery. The mean intestinal volume at time of engraftment and after 10 months was 4.08 cm^3^ (95% CI [3.58–4.58]) and 3.26 cm^3^ (CI 95% [3.23–3.29]), respectively, with a mean shrinkage of 17.3% (range 10.6–23.8%), without any evidence of stenosis. Morphological analysis revealed the progression of the biological material toward a neoformed intestine similar to the native intestine, especially at 8 and 10 months. In a rodent model, we demonstrated that a neointestine, obtained from a biological scaffold showed glucose absorption and a durable increase in diameter.

## Introduction

Extensive intestinal resections can lead to a clinically significant malabsorption syndrome requiring specialized nutritional therapy, known as short bowel syndrome (SBS) [1]. Clinically, an anatomical and/or functional reduction of the bowel absorption capacity occurs due to the loss of significant intestinal segments. SBS usually results from mesenteric thrombosis, Crohn’s disease, trauma or motility disorders in adults and intestinal atresia, gastroschisis and necrotizing enterocolitis in infants [2]. The extent of electrolyte imbalance and nutritional deficiency in SBS depends on either the segment of intestine involved, or on the length and function of the residual intestine [3]. The residual intestine undergoes a compensatory process known as intestinal adaptation that implies bowel lengthening and dilatation to increase the overall surface area, with a marked increase in villus height and crypt depth resulting in a more efficient absorption per square centimeter [4–6]. Early enteral nutrition fosters intestinal remodeling and along with dietary intervention, pharmacological measures and surgical techniques are the bedrock to wean patients off parenteral nutrition and obtain a clinical nutrition autonomy [5, 7–12]. When intestinal adaptation is suboptimal and insufficient to guarantee daily nutrient requirements, patients will suffer from permanent intestinal failure [13]. In this case the standard of treatment remains the optimization of long-term parenteral nutrition and bowel rescue procedures such as Bianchi’s or serial transverse enteroplasty procedure (STEP) procedures, which in large series have shown promising results with a significant reduction in intestinal transplantation [14]. Intestinal transplantation is currently offered only to patients with evolving liver disease, and/or recurrent sepsis from total parenteral nutrition [15].

Recent advances in the field of tissue regeneration are offering promising therapeutic tools for the treatment of SBS [16, 17]. Several reports have described animal models in which the feasibility and efficacy of acellular biologic scaffolds (ABSs) derived from extracellular matrixes of different tissues can promote the growth of neointestine that histologically resembles native intestine [18–25]. These ABSs have been used as serosal patches or tubular structures interposed between intestinal loops to optimize the volume-to-surface-area ratio, increasing the absorption area and thus the residence time of nutrients on intestinal mucosa. While several studies have widely demonstrated the growth of a well-developed three-layered neointestine, there are only a few studies on its actual absorptive capacity [24, 26–29]. Furthermore, engrafted segments, especially those interposed as tubular structures, showed a high incidence of leakage, obstruction, stenosis, and shrinkage, preventing them from being extensively adopted in preclinical studies [18, 19, 21, 22, 24].

The study’s primary endpoint was to investigate the glucose absorptive capacity of the neointestine obtained using a patch of ABS as a scaffold for tissue regeneration in a rat model. The secondary endpoint was to monitor the steadiness of the increase in the engrafted segment area for potential use in the treatment of SBS.

## Methods

Twenty-four adult male Sprague–Dawley rats (Charles River Laboratories, Lodi, Italy) weighing 450–500 g were used in this study. Institutional Review Board (IRB) approval was obtained and all animals were housed in the animal research facility of the University of Verona in accordance with the Committee guidelines for the care of laboratory animals. Rats were fasted for 12 h prior to surgery. All the animals were anesthetized using a mixture of oxygen and isoflurane delivered using an oronasal device. In 12 rats, a median laparotomy was performed exposing the distal ileum, 15 cm proximally to the ileocecal valve. A longitudinal anti-mesenteric enterotomy of 2 cm was performed and a 4-ply 2.2 × 1.5 cm piece of Surgisis^®^ (Cook Medical, Ireland) was engrafted using a 6/0 monofilament running suture. The ABS was kept in saline solution for 15 min before engraftment. Engrafted ileum was accurately measured before and after engraftment. The abdomen was then closed in a single layer with 3/0 silk running suture. The other 12 rats were sham-operated and used as controls. In these rats, a median laparotomy was performed exposing the distal ileum whose diameters were accurately measured before closure. All rats were monitored postoperatively and warmed until fully awakened. Subcutaneous buprenorphine was used as an analgesic for the next 48 h. Animals were then maintained on a liquid diet for the first 24 h and then converted to a full rat chow diet. All rats that underwent the operation were monitored during the study period for body weight and general conditions.

Four rats in the engrafted group and four rats in the sham-operated group were killed at 4, 8, and 10 months postoperatively. Under anesthesia a median relaparotomy was performed. The engrafted ileal segments were identified and measured, and the ileal segments of the sham-operated rats (SORs) were also measured. The engrafted segment was then isolated proximally and distally with silk ties. The same procedure was performed in a segment of distal ileum of the same length in SORs. Vascular arcades proximal and distal to the isolated ileal segment were ligated, while the vascular pedicle supplying the isolated ileal segment was isolated and preserved in both animals.

To investigate the absorptive capacity of glucose in the neointestine we used XenoLight RediJect 2-DG-750 Probe (Caliper Life Sciences, Hopkinton, Massachusetts, USA) (2-NBDG), a near infrared fluorescent probe with peak excitation at 745 nm and peak emission at 780 nm, as a tracer for the evaluation of cellular glucose metabolism [30, 31]. The 18F-FDG analog 2-NBDG has been extensively used for in vivo targeting of cells, such as tumors, that typically exhibit an elevated glucose uptake rate in comparison to surrounding tissue [32, 33]. After exposing the intestine, 500 µl of fluorescent probe diluted 1:5 in a 2 M glucose solution in water was injected into the lumen of the isolated ileal segment in engrafted rats and in SORs, employing a nontraumatic plastic microneedle. Optical images were acquired by an IVIS Spectrum optical imager (Perkin Elmer, Massachusetts, USA). The IVIS Spectrum is usually based on a cooled (− 90 °C) back-thinned, back-illuminated charge-coupled device camera. The average radiance was measured by drawing the region of interest over the images calibrated in radiance units (p/s/cm^2^/sr). Image acquisition, processing and analysis were performed with Living Image 4.5 (Perkin Elmer, Massachusetts, USA). Optical images were acquired in fluorescence mode with the following image parameters: excitation filter 750 nm, emission filter 780 nm, exposure time = automatic, *f* = 2, Binning = 8. Images were acquired before injection and every 15 min after injection of the fluorescent probe for 60 min. During the acquisition phase, the animals were maintained under general anesthesia and hydrated with intraperitoneal delivery of saline solution. Blood samples were obtained by venipuncture at laparotomy and then 30 and 60 min after the injection of the fluorescent probe and assayed with a commercial glucose meter (Freestyle Optium Neo, Abbott Diabetes Care Ltd, Oxon, UK). After 60 min, the isolated ileal segment in both animals was harvested 1 cm proximally and 1 cm distally from the silk ties with its vascular pedicle intact. All specimens were then longitudinally opened in the antimesenteric border, washed three times with physiological solution, dried and acquired again in fluorescent modality. The animals were then killed.

The specimens were then processed for optical microscopy. They were traditionally processed (formalin-fixed paraffin-embedded samples) 34. Tissue blocks were cut into 4 μm sections and stained with hematoxylin–eosin for conventional histological analysis. Alcian-PAS was used to evaluate the mucosal basal membrane, S100 was used to evaluate the bowel innervation, and CD34 and podoplanin were used to evaluate lymphatics and blood vessels. Intestinal components were obtained using Aperio software (at least three fields per sample for each morphologic data). Immunohistochemical analysis was conducted as previously described [35, 36]. Briefly, we used the standard polymer system and peroxidase methods. Heat-induced antigen retrieval for the antibodies S100 (rabbit, 1:3000 dilution) (Dako, Hamburg, Germany), CD34 (clone:QBEND/10, 1:200 dilution) (Leica Biosystems, Newcastle upon Tyne, United Kingdom), Podoplanin (clone:D2-40, 1:50 dilution) (Dako, Hamburg, Germany) was performed with a heated plate and 0.01 mol/l of citrate buffer, pH 8.9, for 15 min. Finally, nuclear counterstaining was performed with hematoxylin for 5 min. All samples were processed using a sensitive ‘Bond Polymer Refine’ detection system in an automated Bond Immunohistochemistry Instrument (Vision-Biosystem, Leica, Milan, Italy). Sections incubated without the primary antibody served as negative controls.

## Statistical analysis

Descriptive statistics were performed to examine any difference in the results between the engrafted animals and controls. Parametric two-tailed Student’s *T* test for statistical validation was used. *P* value was considered statistically significant when < 0.05. Statistical analyses were calculated using SPSS software (IBM, Chicago, Ill, USA) and and MedCalc (v 14.2.1, MedCalc Software, Oostende, Belgium).

## Results

**Fig. 1:**
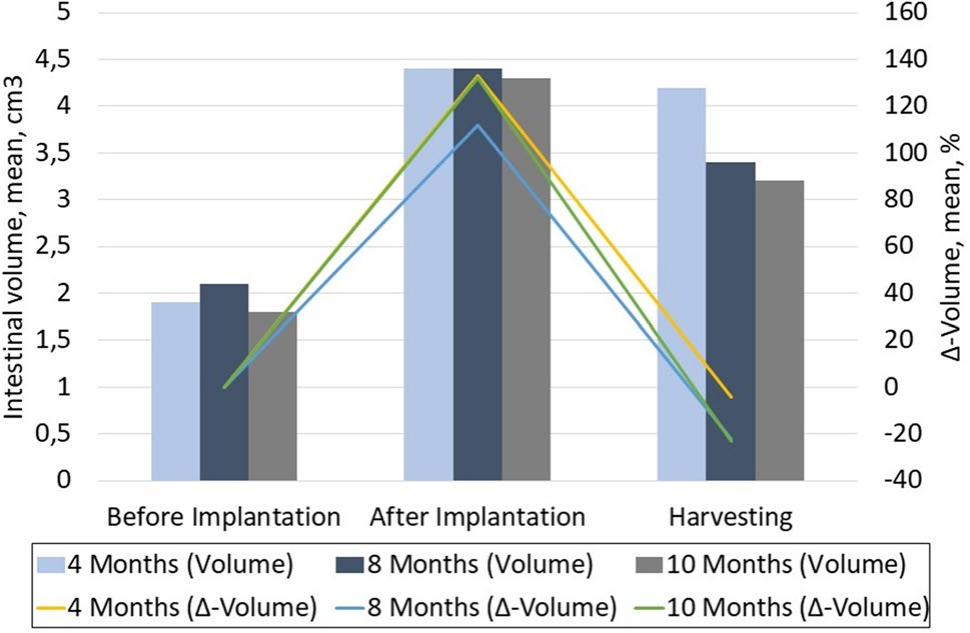
The volume of the engrafted intestinal segments at every time-point in each group of rats. In all engrafted segments, a significant increase in intestinal volume was demonstrated. The mean intestinal volume after 8 and 10 months showed a mean shrinkage of 15.9% (range 9.8–20.4%) and 17.3% (range 10.6–23.8%), respectively, compared to the mean intestinal volume at the time of engraftment. This difference was not statistically significant

All engrafted rats and SORs recovered uneventfully from surgery and both increased their body weight similarly. Four animals in each group were killed as planned at 4, 8 and 10 months. In the engrafted group, the ileal segment with ABS showed a rough serosal surface, and in four cases, mild adhesions between the regenerated bowel and the surrounding tissue were found. In all engrafted segments, an evident increase in intestinal volume was found (Fig. 1). The mean intestinal volume (MIV) of the SORs was 1.75 cm^3^ (95% CI [1.66–1.85]) and was stable over time. In the group of engrafted rats killed at 4, 8 and 10 months, the MIVs at the time of engraftment were 4.27 cm^3^ (95% CI [4.21–4.32]), 4.04 cm^3^ (95% CI [3.77–4.32]) and 4.08 cm^3^ (95% CI [3.48–4.58]), and the MIVs at harvesting were 4.20 cm^3^ (CI 95% [4.18–4.22]), 3.39 cm^3^ (CI 95% [3.25–3.52]) and 3.26 cm^3^ (CI 95% [3.23–3.29]), respectively, without any evidence of stenosis or diverticular formation in any of the engrafted segments. The MIVs after 8 and 10 months showed a mean shrinkage of 15.9% (range 9.8–20.4%) and 17.3% (range 10.6–23.8%), respectively, compared to the MIVs at the time of engraftment. This difference at 8 and 10 months was not significant.

**Fig. 2:**
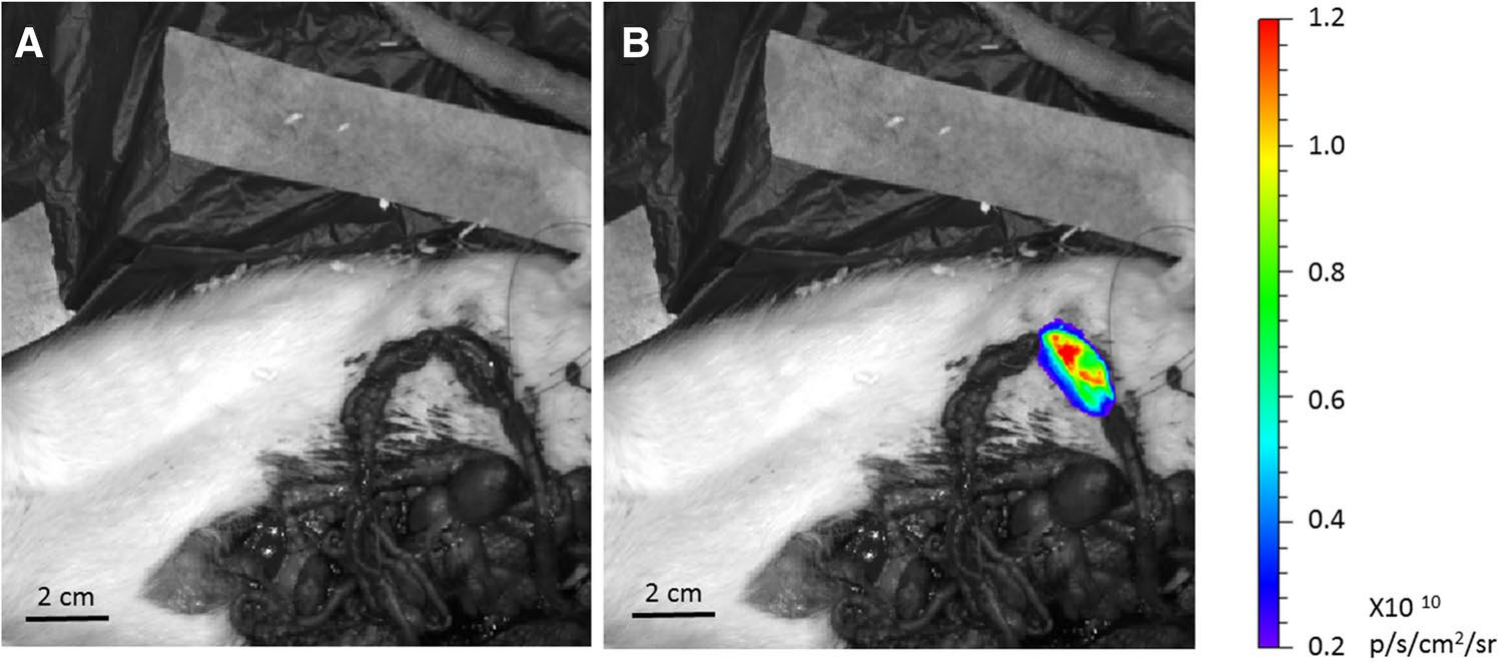
The absence of autofluorescence contamination in the measurements (**A**). In all the rats, fluorescent images superimposed on the anatomical images revealed the presence of the fluorescent 2DG in the tract of the engrafted intestine between the two silk ties after injection (**B**). In the first minutes after injection, fluorescence emission is restricted to the isolated ileal segment

**Fig. 3:**
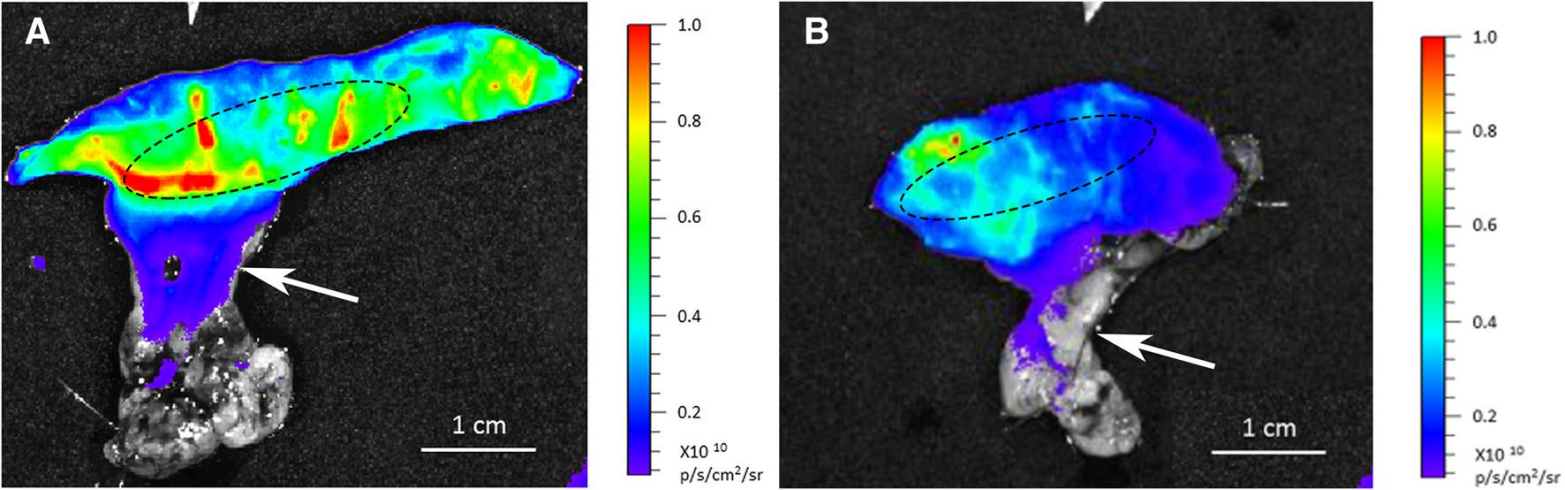
The homogeneous perfusion of the fluorescent 2DG after opening the intestine in engrafted rats (**A**) and SORs (**B**). The segment encircled by the ellipse in **A** represents the ABS, and in **B**, a native intestinal segment of SORs. The fluorescent 2DG uptake of the bioscaffold patch is homogeneous and comparable to that of SORs. In all rats glucose uptake was not restricted to the native and neoformed intestine but was also visible in the preserved mesentery (white arrow) with an initial low fluorescence emission, which became progressively more intense

In all the rats, fluorescent images superimposed on the anatomical images (Fig. 2) revealed the presence of the fluorescent 2-NBDG in the tract of the engrafted intestine between the two silk ties. The fluorescent emission was well visible, and the fluorescence intensity measured before injection was very low, indicating the absence of autofluorescence contamination in the measurements. The power of the signal emitted by the neointestine at 8 and 10 months was comparable to the emission of the ileal segment of SORs (Table 1). The emission of both was stable for the first 60 minutes after fluorescent probe injection. Once opened, intestinal segments of engrafted rats and SORs showed a homogeneous fluorescence emission (Fig. 3). The fluorescent 2DG uptake of the bioscaffold patch was homogeneous and comparable to that of the native intestine of SORs. In all the rats, fluorescence emission was not restricted to the neoformed or to the normal intestine. After 5 min, 2-NBDG diffusion was visible in the preserved mesentery with an initial low fluorescence emission, which became very intense after 60 min.

**Table 1:** Average radiance measured over the images calibrated in radiance units (p/s/cm^2^/sr)

Time	Radiance, mean (IQR)		*P*
	Engrafted	Sham	
4 months	0.08 (1.16) × 10^9^	3.67 (0.62) × 10^9^	**0.02**
8 months	4.56 (0.29) × 10^9^	4.68 (0.49) × 10^9^	0.5
10 months	6.68 (1.03) × 10^9^	6.81 (1.36) × 10^9^	0.7

*IQR* interquartile ranges

**Fig. 4:**
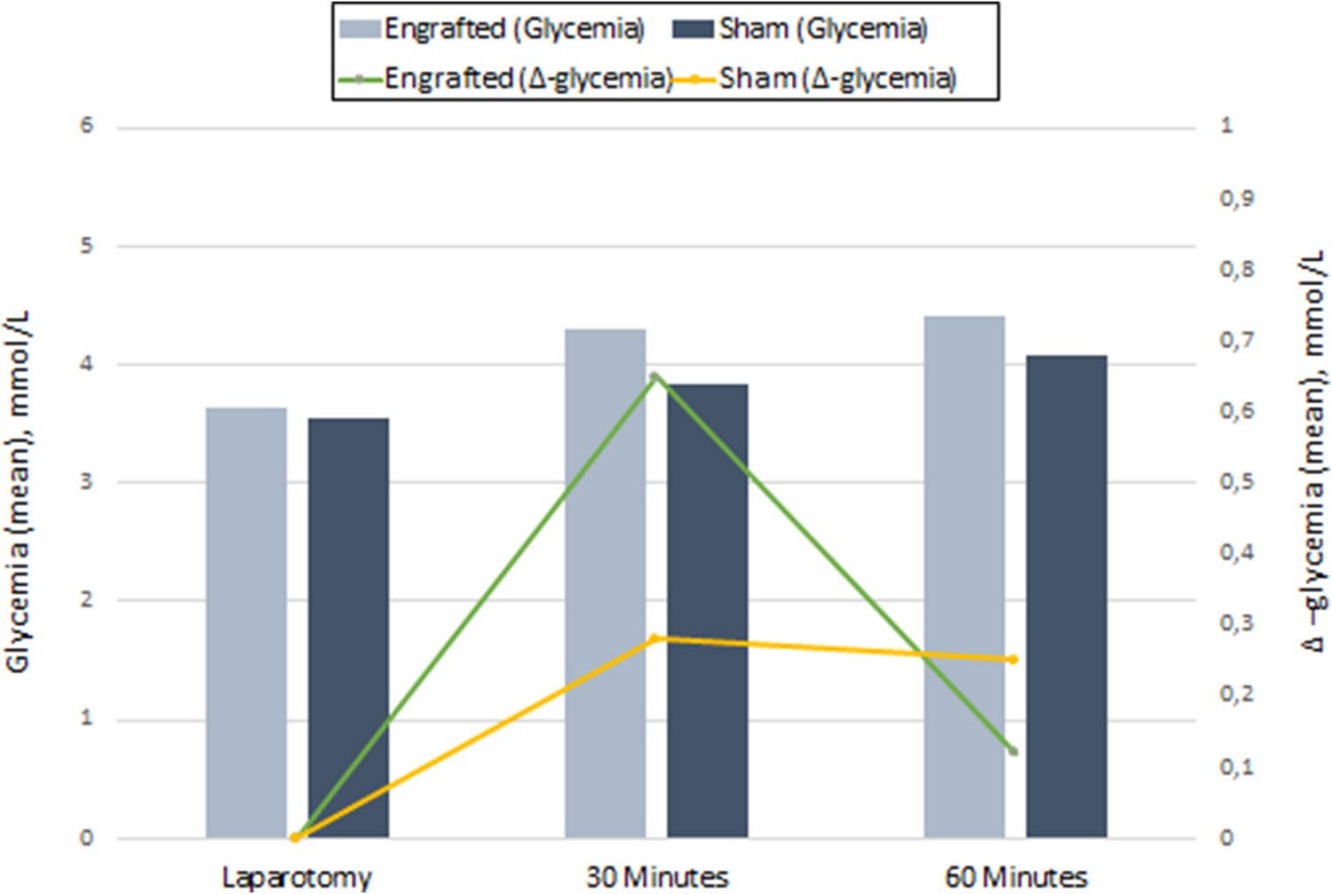
Mean fasting glucose levels and at 30 and 60 min after probe injection. The difference in serum concentration of deoxyglucose analog was higher at the first time point in those animals with the wider absorptive area. For the same reason, this absorption difference gradually diminished at 60 min after glucose administration, as expected

The mean fasting glucose levels in the engrafted rats and SORs were 3.64 mmol/L (95% CI [3.37–3.90]) and 3.55 mmol/L (95% CI [3.22–3.87]), respectively. The mean glucose levels at 30 and 60 min after probe injection were significantly higher in the engrafted rats then in the SORs (4.29 mmol/L (95% CI [4–4.5] vs. 3.83 mmol/L (95% CI [3.54–4.11]), *P* = 0.019), and 4.41 mmol/L (95% CI [4.18–4.6] vs. 4.08 mmol/L (95% CI [3.88–4.34]), *P* = 0.043), respectively. The mean difference between fasting glucose levels and those obtained at 30 and 60 min in engrafted rats and SORs was + 0.65 (range + 0.24/ + 1.45) and + 0.28 (range + 0.16/ + 0.33) (*P* = 0.01), and + 0.12 (range − 0.9/ + 0.33) and + 0.25 (range + 0.12/ + 0.44) (*P* = 0.02), respectively (Fig. 4).

**Fig. 5:**
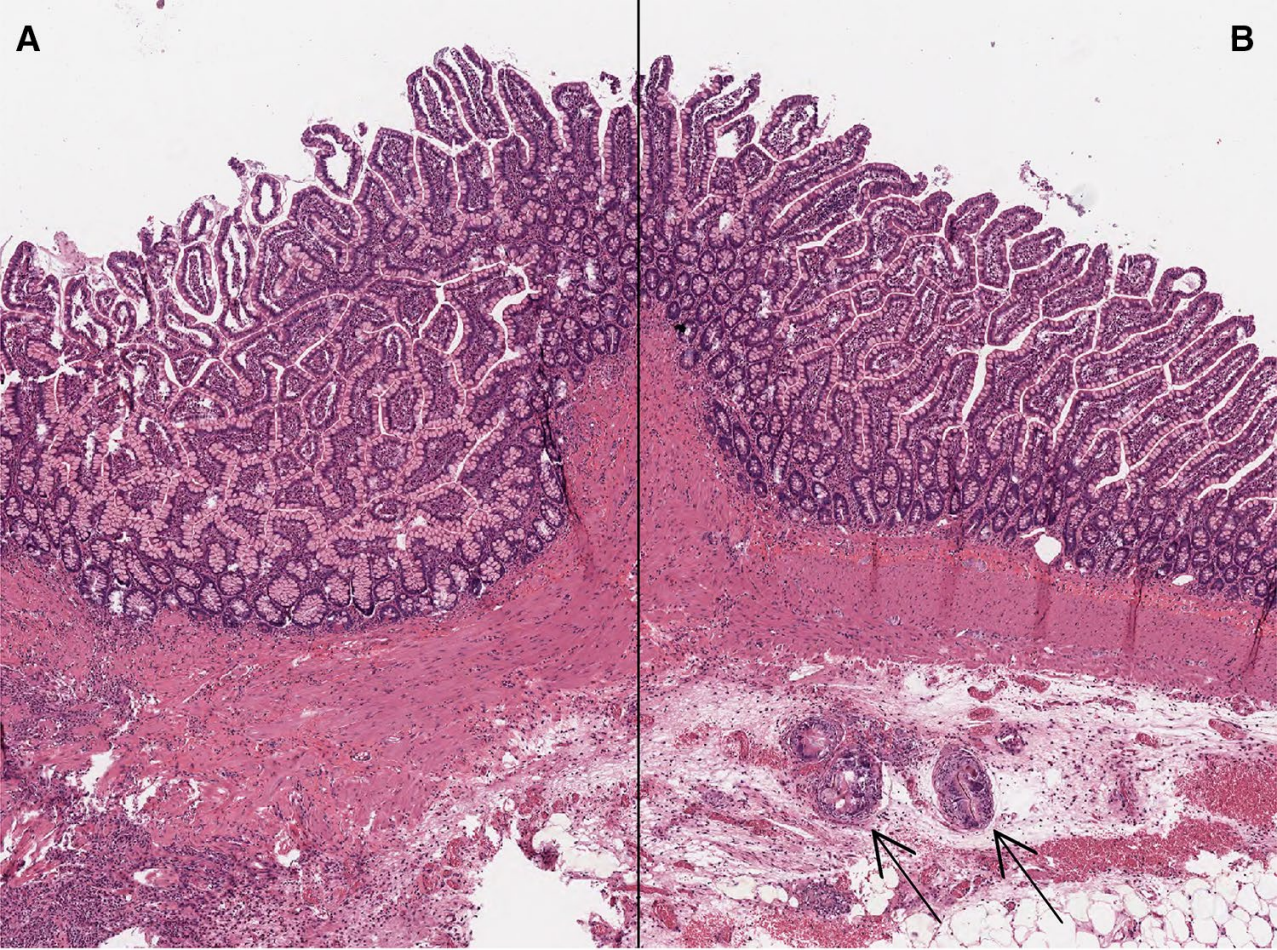
H&E-stained histologic photograph of the native intestine (**A**) and of neoformed intestine (**B**) at 10 months. The neoformed intestine has a similar architectural structure and cell morphology as the native control intestine. Arrows indicate foreign body granulomas due to incomplete ABS integration (original magnification: 4×)

Morphological analysis at 4, 8 and 10 months revealed ABS progression toward a neoformed intestine similar to the native intestine (Fig. 5). The neoformed intestine showed three well-developed layers, especially at 8 and 10 months. On the serosal side of the engrafted segment, we demonstrated some foreign body granulomas due to incomplete integration of the ABS. The three layers were already present in the rats killed at 4 months, although the muscularis propria contained disorganized smooth muscle cells with a haphazard arrangement in an inflammatory environment. In turn at 8 and 10 months, the muscularis propria showed a well-developed longitudinal and circular muscle layer. At each stage of development, there was no significant difference in the thickness of the mucosa and muscularis propria or in the crypt depth and villus height between the neointestine and the normal intestinal wall. Immunohistochemical staining for the protein S-100 highlighted a well-developed submucosal and myenteric plexus in all engrafted segments with evidence of ganglia and fibers especially at 8 and 10 months. A rich neovascularization supported the neoformed intestine with a well-developed network of lymphatic vessels.

## Discussion

Our study showed that neointestines obtained from a Surgisis patch offer evidence of in vivo glucose absorption. We also showed that the engrafted segment undergoes a physiologically expected amount of shrinkage (17.3%) that becomes stable over time, maintaining an advantageous volume-to-surface-area ratio.

In this rodent model, Surgisis promoted optimal mucosal epithelial regeneration accompanied by a complete neovascularization. From the earliest stages, a normal small intestinal architecture, including an intact mucosa and submucosa, was documented. At 4 months, the muscularis propria was slightly disorganized and lacked the circular and the longitudinal layers of smooth muscle fibers. At 8 and 10 months, however, it was fully regenerated showing a circular and longitudinal layer of smooth muscle fibers with a fully developed nervous network. The lack of a fully developed muscularis propria did not cause any motility problems as reported by previous reports [22, 24]. This problem seems to occur mostly in cases of interposed tubular grafts [25], but in this model of intestinal regeneration, the persistence of the whole native muscularis propria preserved physiological motility with a good intestinal transit.

A near-infrared fluorescent deoxyglucose analog, 2-NBDG, has been used to demonstrate the glucose absorptive capacity of the neointestine. Optical molecular imaging is a newer technology with many advantages over other imaging approaches, such as real-time image acquisition, low relative cost, high spatial resolution, and lack of ionizing radiation [30–33]. The use of ^18^F-FDG analogs such as 2-NBDG relies on the fact that these molecules are first recognized and transported into cells by specific glucose transporters (GLUTs) located on the cell membrane [34]. In cancer cells, 2-NBDG, similar to ^18^F-FDG, is then phosphorylated by hexokinase to ^18^F-FDG-6-phosphate for glycolytic processing. Because its phosphorylated form is not a substrate for the glucose-6-phosphate isomerase, most of the labeled analog accumulates in cells [35]. In turn, in normal cells 2-NBDG, similar to ^18^F-FDG, is significantly metabolized to subsequent oxygenation products such as 2-^18^F-fluoro-2-deoxy-6-phospho-D-gluconate (^18^F-FD-PG1) and 2-^18^F-fluoro-2-deoxy-6-phospho-D-gluconolactone (^18^F-FD- PGL) [36], which can be released from cells and can be visible in the liver 45 min after injection [37]. Our model took advantage of 2-NBDG pharmacokinetics highlighting the active glucose uptake of the neoformed and native intestine immediately after injection and its release in the mesenteric circulation already 5 min after injection. Near-infrared fluorescence imaging demonstrated that the intensity of the signal emitted by the neointestine was comparable to the emission of the native intestine of controls, showing no difference in the glucose uptake. The fluorescence emission of the neointestine was stable and similar over time to that of the native intestine, showing a fully developed absorptive capacity of glucose.

To our knowledge, this is the first direct evidence of the absorptive capacity of a neoformed intestine. Previous studies on rodent models have shown only indirect evidence of nutrient absorption by a neoformed intestine. Cicalese et al. [24] demonstrated the presence of structural elements that are deputed to glucose absorption, by emphasizing the similarity of the cell architectural structure between the neoformed and native intestine. They observed an increase in intestinal absorption and urinary excretion in the neoformed intestine 2 h after intraluminal administration of D-xylose versus native intestine at 12 weeks post engraftment. The neointestinal segment showed a D-xylose absorption of approximately 50% more compared to the native intestine of controls. Our study confirmed these findings showing a physiological increase in glucose serum levels in both the engrafted and control groups at 30 and 60 min after the deoxyglucose analog administration. In engrafted rats, this increase at 30 min was approximately double compared to that in the SORs. This interesting finding likely reflects the largest absorption surface of the neoformed intestine which is approximately double that of controls. Given the same intraluminal concentration, the difference in serum concentration of glucose was higher at the first time point in those animals with the wider absorptive area. For the same reason, this absorption difference gradually diminished at 60 min after glucose administration, as expected.

In this study, we monitored possible modifications of the increased absorptive area of the neoformed intestine over time, and we found a mean shrinkage of 17.3% after 10 months of engraftment, without any evidence of stenosis. The Surgisis shrinkage over time has already been reported by other authors [22]. Surgisis is a biological graft extracted from porcine small intestinal submucosa [38]. When used as a biological scaffold, Surgisis is usually associated with optimal tissue remodeling, the presence of well-organized tissue with almost complete resorption and a variable shrinkage percentage. The process of remodeling via gradual replacement of Surgisis with organized tissue requires a certain amount of scaffold resorption that leads to a shrinkage and shortening of the engrafted intestinal segment.

Ansaloni et al. reported a mean shrinkage of 30% of Surgisis while highlighting the full development of the neointestine in a rodent model [22]. They interposed a 3-cm tubular Surgisis graft in the middle of an isolated ileal loop with a double end-to-end anastomosis. Although ileal loop vascularization has been preserved, the insufficient blood supply to the midportion of the graft could explain such a shrinkage extent. Other similar reports seem to confirm that a good vascular supply is critical for balanced growth of the neointestine especially at the earliest phase of ABS remodeling [17, 22, 40, 41]. Strictures and important shrinkages were not found in ABS antimesenteric patches, probably because every part of the patch is close to the bowel's intact edges for its whole length and could take advantage of the good vascular supply of the surrounding intestine [24, 25]. Angiogenic factors, along with the growth factors, mitogenic factors, and chemotactic cytokines that are usually preserved in extracellular matrixes play a crucial role for neovasculogenesis. This process is enhanced by the recruitment of endothelial cells and endothelial progenitor cells that can induce the development of a dense vascular network that is already visible in the early phases of remodeling [41].

To define this important aspect of the remodeling process we decided to kill the rats after a longer period compared to other studies where the animals were killed few months after engraftment [22, 24]. We did not find any significant difference in the absorptive area shrinkage between 8 and 10 months. This finding could suggest that the Surgisis remodeling process is almost complete at 10 months. It is reasonable to argue that after this time the remodeling process could undergo marginal modifications leaving unchanged the net absorption area of the native intestine and the neoformed intestine could be used in intestinal lengthening procedures without any substantial reduction in volume.

The process of intestinal adaptation usually follows extensive intestinal resections to achieve a more advantageous volume-to-surface-area ratio of the residual intestine to obtain clinical nutrition autonomy [3, 4]. For this reason, intestinal lengthening procedures are, therefore, an interesting approach to SBS. They are basically represented by Bianchi’s and by the STEP procedures [11, 12, 42, 43]. Both methods can double the bowel’s length, but this goes to the detriment of the diameter that is usually halved, leaving the net absorption area unchanged. For these reasons, these procedures have shown promising results but until now have only limited curative potential, and they are considered by many only as a bridge to intestinal transplantation [14, 38]. An efficient surgical approach should in fact promote a simultaneous and immediate increase in the overall intestinal absorptive area, thus mimicking the process of intestinal adaptation. The proposed technique of antimesenteric patching with ABS seems to be a valuable tool to lengthen the residual intestine since it offers an immediate and advantageous volume-to-surface-area ratio, avoiding complications such as shrinkage and stricture [22, 39, 40].

The present study has not negligible limitations. The distal ileum was chosen over duodenum and jejunum as the preferential site of engraftment because it was more technically suitable for the surgical engraftment of the biological scaffold due to its size. It is known that the ileal absorption of glucose is smaller when compared to duodenum and jejunum because the transmembrane transporters SGLT1 and GLUT2 are present in low quantities, especially in the distal ileum [44]. However, the distal ileum has shown a great adaptability in glucose absorption after proximal ileal resections [45]. Although this represents the longest follow-up available in the literature evaluating the remodeling of a neointestine obtained from a biological scaffold in a rodent model, the duration of the study may not be sufficient to monitor the steadiness of the neointestinal volume achieved, although the volume changes after the 8 month of follow-up are not statistically significant.

## Conclusions

In a rodent model, we demonstrated that the neointestine, obtained from ABS, shows glucose absorptive capacity and a durable increase in volume with a more advantageous volume-to-surface-area ratio. We believe that this model is simple and readily translatable to clinical application in patients suffering from SBS.

## Data Availability

N/A.

## Code availability

N/A.
